# Supplementary material for: Spoken Expressive Vocabulary in 2-Year-Old Children with Hearing Loss: A Community Study
Source: Children (Basel). 2023 Jul 14;10(7):1223. doi: 10.3390/children10071223 (PMC10377817; doi:10.3390/children10071223)
Supplement: Supplementary file 1 [file children-10-01223-s001.zip › VicCHILD Expressive Vocab Figure S1.pdf]

(a)

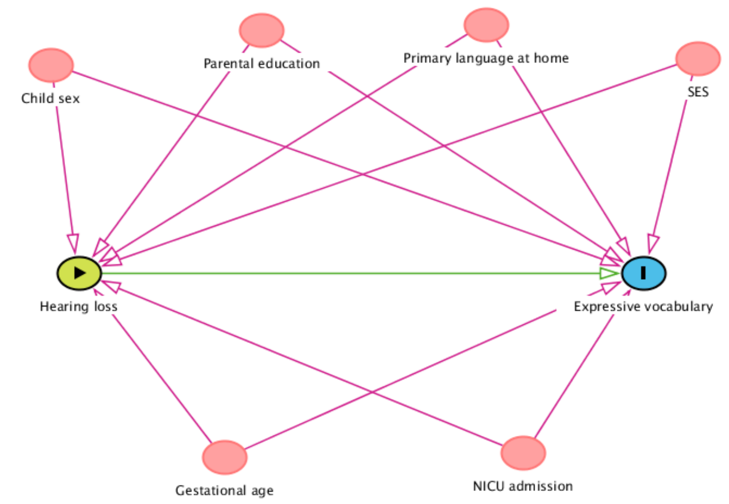

(b)

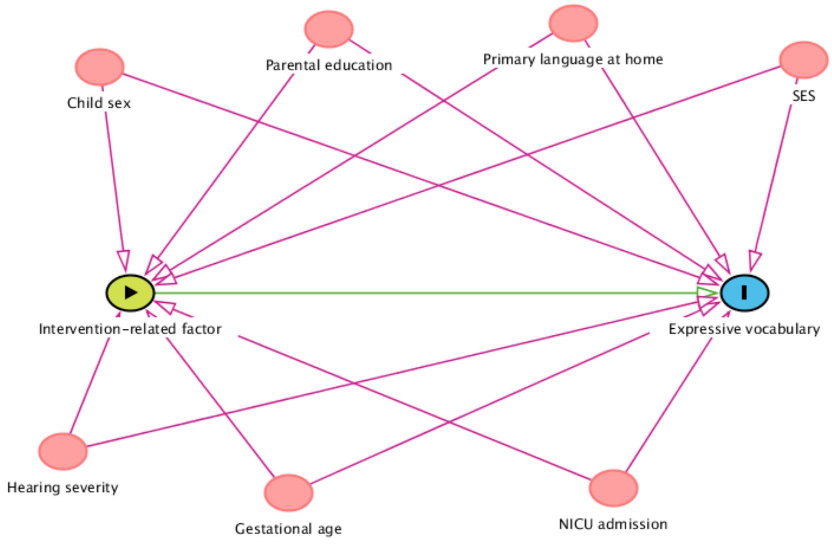

Figure S1. Statistical approach with directed acyclic graphs of (a) the relationship between hearing loss and expressive vocabulary at age two years (used for aim 1), and (b) the relationship between intervention-related factors and expressive vocabulary at age two years (used for aim 2). Intervention-related factors were considered separately within each model.
