# Supplementary material for: Spoken Expressive Vocabulary in 2-Year-Old Children with Hearing Loss: A Community Study
Source: Children (Basel). 2023 Jul 14;10(7):1223. doi: 10.3390/children10071223 (PMC10377817; doi:10.3390/children10071223)
Supplement: Supplementary file 1 [file children-10-01223-s001.zip › VicCHILD Expressive Vocab Table S1.pdf]

Table S1. Frequencies of hearing loss across exposures used for aim 2.

	Unilateral hearing loss				Bilateral hearing loss			
	Mild	Moderate	Severe	Profound	Mild	Moderate	Severe	Profound
**Device used at survey**								
No	6	17	15	26	18	3	0	1
Yes	6	5	8	10	39	70	38	40
**Age device first fitted**								
≤ 3 months	1	1	0	6	12	40	27	27
3.1 - 6 months	1	4	6	4	8	17	6	10
> 6 months	4	4	5	5	17	14	4	4
**Average device use**								
Always	3	0	2	1	16	37	28	30
Sometimes/often	1	4	3	6	12	19	7	10
Never/rarely	8	18	18	27	28	13	2	1
**Age EI program enrolment**								
≤ 3 months	0	0	2	2	3	13	17	14
3.1 - 6 months	2	3	0	4	13	26	12	14
> 6 months	1	2	6	7	19	28	9	11
